# P-772. Retrospective, Multicenter Evaluation of Initial Therapy for Suspected Urinary-Source Sepsis in the Absence of Risk Factors for Ceftriaxone Resistance: Ceftriaxone versus Cefepime or Piperacillin-tazobactam

**DOI:** 10.1093/ofid/ofaf695.983

**Published:** 2026-01-11

**Authors:** Harrison Phan, Alice M Landayan, Lourdes R Menendez Alvarado, Lee Amaya, Wilbert Fuerte, Timothy Gauthier

**Affiliations:** Baptist Health, West Covina, California; South Miami Hospital, South Miami, FL; Baptist Health, West Covina, California; Miami Cancer Institute Baptist Health, Miami, Florida; Homestead Hospital, Homestead, Florida; Baptist Health South Florida, Miami, FL

## Abstract

**Background:**

Initial antibiotic selection for treatment of urinary-source sepsis is of considerable importance for antimicrobial stewardship programs. Use of cefepime (FEP) or piperacillin-tazobactam (PTZ) instead of ceftriaxone (CRO) may be undesirable in the presence of risk factors for CRO-resistant pathogens or absence of severe illness. This study aims to describe practices and compare clinical outcomes amongst patients who received CRO versus FEP or PTZ as empiric therapy for suspected urinary-source sepsis, in the absence of risk factors for CRO resistance.

**Figure:**
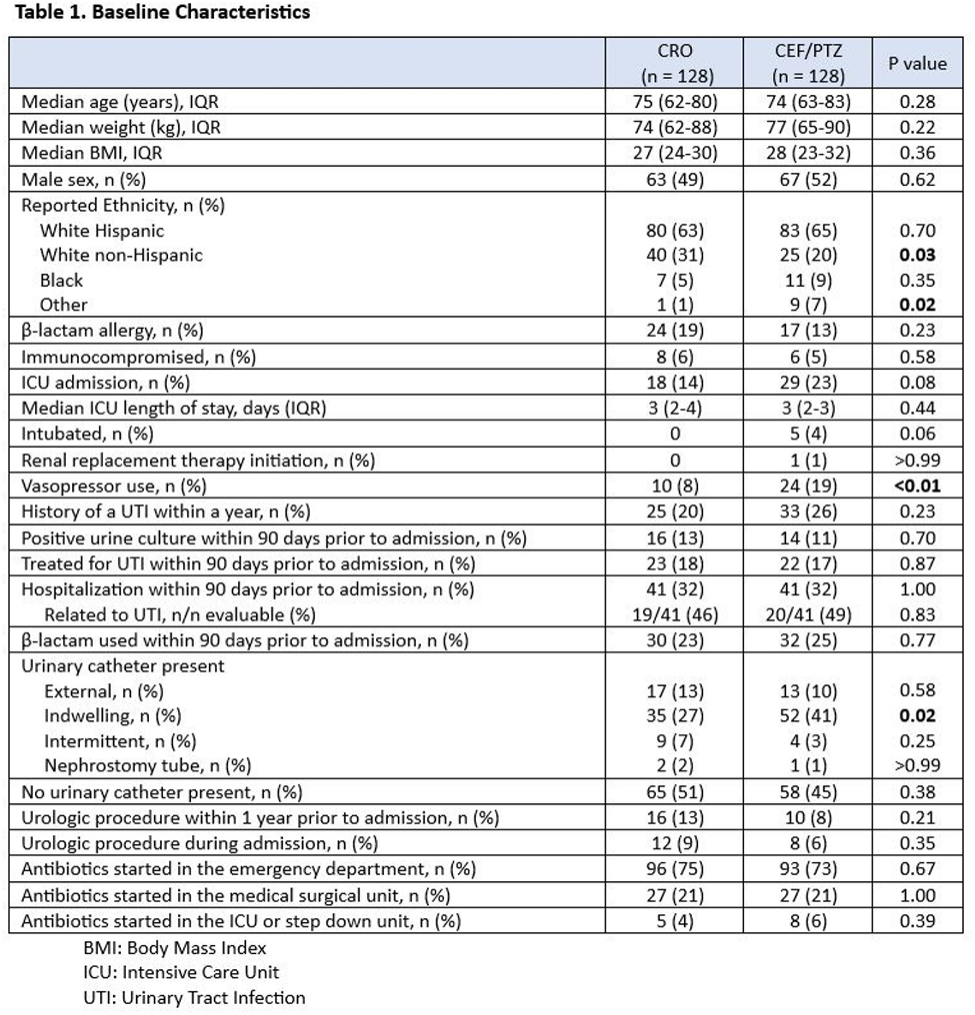

**Figure:**
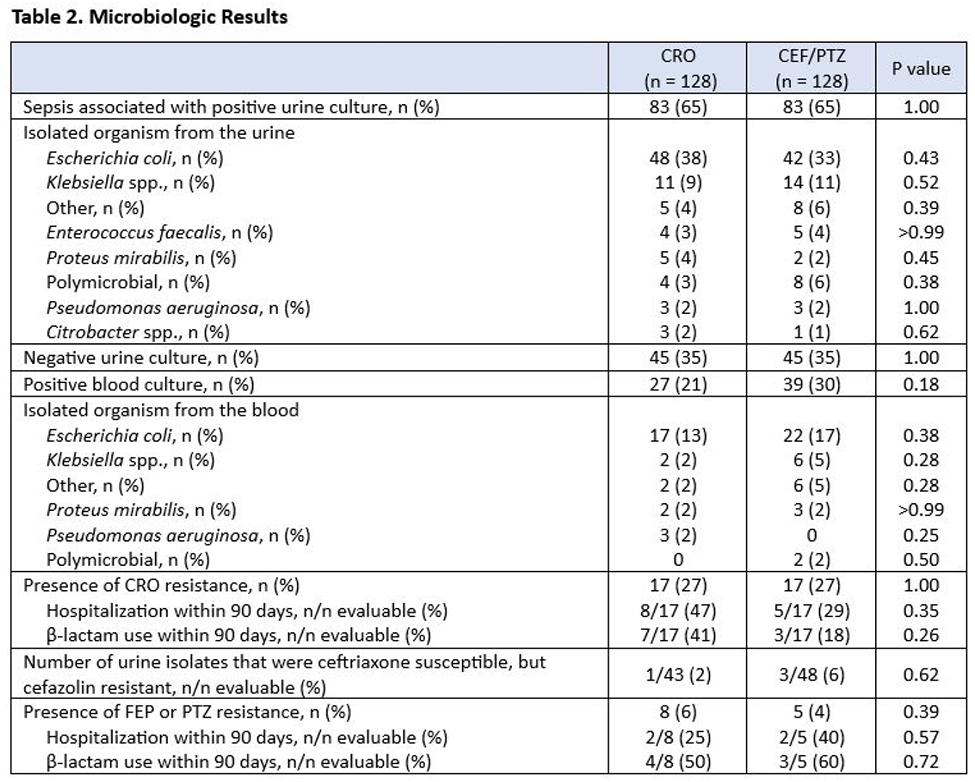

**Methods:**

This retrospective, multicenter, quasi-experimental study included patients ≥ 18 years admitted with suspected or confirmed sepsis from urinary source who received CRO, FEP, or PTZ as initial empiric therapy. Patients were included if they received one of the targeted antibiotics between November 2023 through November 2024. Patients were excluded if they received antibiotic treatment for less than 48 hours and had presence of risk factors for CRO-resistance. The primary outcome was 30-day all-cause in-hospital mortality. Secondary outcomes included change from empiric regimen (escalation or de-escalation of therapy), antibiotic duration, hospital length of stay, 30-day infection-related in-hospital mortality, and 30-day readmission due to clinical failure. Institutional Review Board approval was granted.

**Figure:**
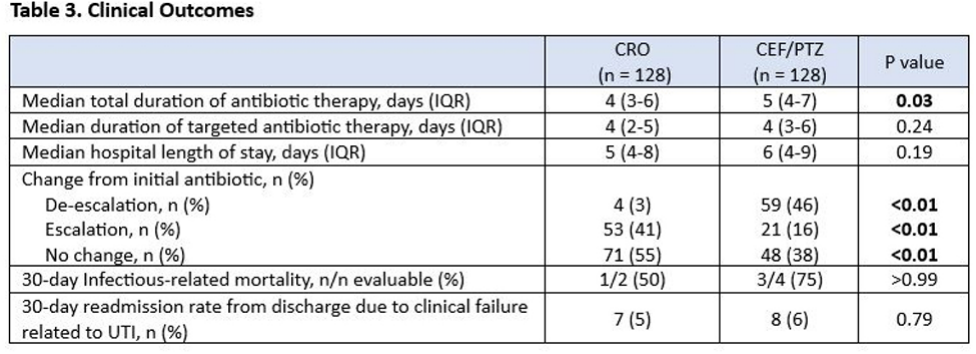

**Results:**

A total of 371 patients were screened, of which 115 patients were excluded. The three most common reasons for exclusion were receiving treatment for less than 48 hours (n=56), CRO resistance within the past 3 months (n=26), and lack of documentation indicating sepsis from urinary source in the physician note (n=20). Among 256 included patients, 128 received CRO, 64 received FEP, and 64 received PTZ. Demographics are displayed in Table 1. Thirty-day all-cause mortality occurred in 2 (2%) patients in the CRO arm and 4 (3%) in the FEP/PTZ arm (p = 0.67). Other secondary outcomes are summarized in Table 2.

**Conclusion:**

FEP or PTZ compared with CRO prescribed as initial therapy for urinary-source sepsis did not improve clinical outcomes for patients without risk factors for a CRO-resistant organism.

**Disclosures:**

All Authors: No reported disclosures

